# American Congress on Tuberculosis—Session of 1903-4

**Published:** 1903-08

**Authors:** 


### American Congress on Tuberculosis—Session of 1903-4.

*Honorary Presidents.—Lay:* Hon. John Hay, Gen. Russell A. Alger, Hon. ex-Judge A. H. Dailey, Hon. Judge C. G. Garrison, Hon. The Earl of Minto.

*Medical:* Prof. J. G. Adami, M. D., Dr. A. N. Bell, Prof. Charles H. Hughes, Gen. Presley M. Rixie, Gen. Nicholas Senn.

*Council.*—Moritz Ellinger, Esq., Chairman; J. Mount Bleyer, M. D., New York City; A. P. Grinnell, M. D., Vermont; H. Edwin Lewis, M. D., Vermont; Richard J. Nunn, M. D., Georgia; W. F. Drewey, M. D., Virginia; M. K. Kassabian, M. D., Pennsylvania; J. W. P. Smithwick, North Carolina.

*Officers.*—President, E. J. Barrick, M. D., Toronto, Ontario; First Vice-President, F. E. Daniel, M. D., Austin, Texas; Second Vice-President, L. Bradford Prince, Santa Fe, New Mexico; Third Vice-President, Dr. Charles K. Cole, Helena, Montana; Fourth Vice-President, Dr. Sofus B. Nelson, Pullman, Wash.; Fifth Vice-President, Dr. A. M. Linn, Des Moines, Iowa; Secretary, Samuel Bell Thomas, 290 Broadway, New York; Treasurer, Clark Bell, 39 Broadway, New York.

New York, July 13, 1903.

*To Officers and Members of State Medical Associations or Other Medical Bodies or Associations Interested in the Prevention of Tuberculosis.*

Gentlemen: The Governing Council of the American Congress on Tuberculosis have authorized and directed the undersigned to invite each member of your body to co-operate with this body in a congress to be held at St. Louis in 1904, and if you will contribute a paper to be read before the body or any of its sections, to send the title of the same to the undersigned.

We have also been instructed to ask your organization to appoint at least three delegates to represent your association at such congress, and advise us of the names and addresses of such delegates. An early reply will be appreciated.

Respectfully yours,
E. J. Barrick, M. D., President,
Toronto, Ontario.

Samuel Bell Thomas, Secretary,
290 Broadway, New York City.

---

The following has also been issued by the Congress:

New York, July 14, 1903.

Dear Colleague: At a meeting of the Council of the American Congress on Tuberculosis, a resolution was adopted, unanimously fixing the annual dues of the Congress of 1903 at the nominal sum of $1.00, from June 4, 1902, to June 10, 1903.

The treasurer was ordered to send out bills for the annual dues to every enrolled member of the Congress of 1902. If you wish to remain a member of the Congress for 1903, please remit your dues, $1.00, and your name will be entered on the roll of members of the Congress of 1903. Those who prefer not to be so enrolled will advise the undersigned or the secretary.

You are reminded that by action of the council accepting the offer of the *Medico-Legal Journal,* it was provided, that:

1. The *Medico-Legal Journal*, Vol 21, commencing June number, 1903, will be sent to every member or delegate to the Congress of 1902, or to any member, at half price, $1.50, payable in advance.

2. That the *Medico-Legal Journal* will send, when it is completed, the Bulletin of the Congress of 1902 to every member or delegate not enrolled, nor a present subscriber, at half price, $1.50, if paid in advance, which will include membership in the Congress of 1903, by action of the Council of the American Congress of 1903.

Respectfully yours,
CLARK BELL, Treasurer,
American Congress on Tuberculosis, 39 Broadway, New York.

